# Supplementary material for: Probabilistic forecasting of monthly dengue cases using epidemiological and climate signals: A BiLSTM-Negative Binomial Model versus Mechanistic and Count-Model Baselines
Source: PLOS Glob Public Health. 2026 Mar 27;6(3):e0005404. doi: 10.1371/journal.pgph.0005404 (PMC13029795; doi:10.1371/journal.pgph.0005404)
Supplement: S1 Table — Summary of final model configurations used in the main experiments (e.g., GLM covariates/penalty if any, INGARCH order and link, renewal kernel and seasonal Rt specification, BiLSTM architecture/training settings, and calibration settings). (Provided in a separate upload.). (DOCX) [file pgph.0005404.s007.docx]

# **S2 Table.** BiLSTM-NB training configuration and hyperparameter audit (leakage-safe).

**Component Setting Value(s) How chosen Notes**

Data split Time-based 80/20 Fixed a priori Train first 80%, validate last

20%

Lookback window Sequence length *W* = 12 Fixed a priori Captures annual seasonality Targets Horizons *h* = 1*,* 2*,* 3 Fixed a priori Multi-horizon output Inputs (sequence) Cases history past *W* values Fixed a priori No future leakage

Inputs (aux) Seasonality sin12, cos12 Fixed a priori Month-of-year harmonics

Inputs (aux) AR lags lag1, lag2, lag3, lag12

Inputs (aux) Climate lags precip*t−*1, temp*t−*1, rh*t−*1 (max 3)

Scaling Standardization z-score (climate only)

Fixed a priori Also used in AR-skip Fixed a priori lag0 excluded; leakage-safe

Fixed a priori Count lags left unscaled

Model core BiLSTM layers 2×BiLSTM(32) Fixed a priori First returns sequences Fusion Concatenation BiLSTM + aux Fixed a priori

Dense head Hidden layer 64 (ReLU) Fixed a priori L2=10*^−^*^4^, Dropout=0.2 AR skip Linear map Dense(3) Fixed a priori Additive AR baseline

Likelihood Distribution NB2 Fixed a priori Proper discrete probabilistic

output

Dispersion Bounds *α* ∈ [10*^−^*^4^*,* 2*.*0] Fixed a priori Prevents degenerate variance Optimizer Adam lr=10*^−^*^3^ Fixed a priori clipnorm=1.0

Training budget Epoch cap 150 Fixed a priori Batch size Mini-batch 16 Fixed a priori

Early stopping Patience 12 Fixed a priori Restore best weights

LR schedule ReduceLROnPlateau factor=0.5, pa-

tience=6

Fixed a priori min lr=5 × 10*^−^*^5^

Randomness Seeds 5 (1234–1238) Fixed a priori Ensemble for robustness Ensemble Mixture Equal-weight Fixed a priori Used for scores/quantiles Calibration Isotonic fit first 60% Fixed a priori Avoids look-ahead; applied to

mixture CDF

Selection criterion Best epoch min val NLL Validation-based Determined by early stopping
