# Supplementary material for: Probabilistic forecasting of monthly dengue cases using epidemiological and climate signals: A BiLSTM-Negative Binomial Model versus Mechanistic and Count-Model Baselines
Source: PLOS Glob Public Health. 2026 Mar 27;6(3):e0005404. doi: 10.1371/journal.pgph.0005404 (PMC13029795; doi:10.1371/journal.pgph.0005404)
Supplement: Attachment — Submitted filename: Response_to_Reviewers.pdf [file pgph.0005404.s009.pdf]

Reviewer #1

Comment R1 (overall assessment and requested improvements): *The reviewer finds the study rigorous and technically sound, but notes three main areas to strengthen: (i) improve accessibility by reducing/relocating heavy technical exposition in the main text; (ii) better justify the mechanistic renewal-model assumptions, especially the short fixed kernel; and (iii) expand discussion of generalizability (single-city, monthly cadence, climate treatment) and how these choices may affect conclusions.*

Response: We thank the reviewer for the careful reading and the positive assessment of the study's rigor and operational relevance. We agree that improving accessibility, clarifying the mechanistic baseline assumptions, and strengthening the generalizability discussion will increase the paper's impact for both technical and public-health audiences. In the revision, we streamlined the main narrative, added clearer justification and limitations for the renewal-kernel choice (and why monthly aggregation constrains mechanistic realism), and expanded robustness/generalizability analyses and discussion to better contextualize the horizon-dependent results.

What we changed:

- **Accessibility/readability :** We edited the main text to reduce dense mathematical detail where it was not essential for interpretation, and moved selected technical derivations/extended implementation details to the Supporting Information, while adding brief intuitive explanations in the main text for practitioners.
- **Mechanistic renewal model justification:** We clarified the rationale for the renewal baseline at monthly cadence and explicitly discussed how a short, fixed kernel may be misspecified for dengue generation intervals when data are aggregated monthly. We also added a sensitivity analysis exploring alternative/longer kernel specifications to assess how kernel choice affects probabilistic accuracy, calibration, and sharpness.
- **Climate treatment:** We strengthened justification for the "light climate" design as a real-time feasibility constraint and added text noting exploratory considerations (and the leakage/latency risks) of larger feature sets and longer lag structures in operational settings.
- **Generalizability/distribution shift:** We added an era-based evaluation (restricted to 2021–2024) to probe temporal transportability under potential distribution shift, and we expanded the Discussion to more explicitly address limits to geographic transfer, monthly cadence effects, and implications for outbreaks and mechanistic/hybrid models.
- **Outbreak interpretability:** We added a regime-stratified (outbreak vs non-outbreak) analysis to clarify how model behavior changes under high-incidence targets, emphasizing calibration-sharpness trade-offs and upper-tail miss behavior rather than coverage alone.

Where:

- Accessibility edits: Section [Methods/Results], pp. [6-13;13-21] and Supporting Information Section [SI], p. [24-25].
- Renewal model justification + kernel sensitivity: Section [Methods/Results], p. [11;].
- Climate feature-set justification + ablation: Section [Results: Climate experiment], p. [16-18] and Table [5], Figures [7-9]; p. [18].
- Regime-stratified outbreak analysis: Section [Results: Outbreak vs non-outbreak], p. [18-20] and Table [6]/Figure [10], p. [19].
- Era-based generalizability evaluation (2021–2024): Section [Results: Generalizability across time], p. [19] and Table [7-8], Figures [11-12]; p. [20-21].

Reviewer #2

Comment 1 (Abbreviations in the Abstract: PIT). Reviewer comment: What does PIT stand for? Please avoid abbreviations in the abstract.

Response: We agree and revised the Abstract to avoid unexplained abbreviations. We now write *probability integral transform* in full the first time it appears (and/or rephrased to remove the acronym entirely).
What we changed: Replaced "PIT diagnostics" with "probability integral transform diagnostics" (or removed the acronym).
Where: Abstract, p. [1], lines [12-15].

Comment 2 (Additional analyses: serial-interval kernels/sensitivity checks). Reviewer comment: Provide additional analysis, such as experimenting with alternative or longer serial-interval kernels, or sensitivity checks (different window lengths, or finer temporal resolution if possible).

Response: We agree that additional sensitivity checks improve robustness and interpretability. We added a renewal-model sensitivity analysis varying the serial-interval kernel specification (including longer kernels) and summarized the impact on mean log score, calibration (coverage), and sharpness (interval width) under the same rolling-origin protocol. Where finer temporal resolution was not available, we explicitly note this constraint and focus on kernel and evaluation-design sensitivity at the monthly cadence.
What we added: (i) Alternative/longer renewal kernels; (ii) summary tables/figures comparing performance changes across kernels.
Where: Section [Sensitivity analyses / Renewal model sensitivity], p. [11]; Table/Figure [3; 2-4], p. [14-17].

Comment 3 (Justify the small climate feature set; mention exploratory work with larger sets). Reviewer comment: Please justify the small climate feature set, mentioning any exploratory work with larger sets.

Response: We agree and clarified that the "light climate" design is a deliberate operational constraint: we restrict to a minimal set of lagged climate covariates that are plausibly available at issue time, to avoid leakage and reduce failure modes from latency and missingness in real-time feeds. We added text summarizing exploratory considerations with larger climate sets/lag structures and explain why we did not adopt them for the primary, deployment-oriented evaluation (and, where applicable, we summarize any exploratory findings in the Supporting Information).

What we changed: Expanded justification of the light-climate choice; added/expanded the climate ablation discussion; optionally added a brief exploratory note on larger sets and why excluded from the main protocol.
Where: Section [Results: Climate signal contribution: the "light climate" experiment], p. [16-17]; Table [5]; p [17].

Comment 4 (Missing-data handling subsection: imputation details and sensitivity). Reviewer comment: Add a clearly labelled missing-data handling subsection specifying the imputation method, number of imputed months, how used in training/evaluation, and any sensitivity.

Response: Response: We agree and have added a clearly labelled Missing data handling subsection to make the preprocessing pipeline fully auditable. After aligning the dataset to a complete monthly calendar (Jan 2015–Dec 2024), we verified the completeness of the dengue outcome and the selected "light" climate covariates (precipitation, temperature, relative humidity). In this study dataset, there were no missing months and no missing values in the outcome or selected covariates; therefore, no imputation was required or performed, and 0 months were imputed. Consequently, training and evaluation used the full aligned series without missingness-related exclusions. We additionally included an explicit operational note describing leakage-safe handling procedures for climate-covariate missingness in prospective deployments (fold-wise imputation only, with targets never imputed), although this sensitivity does not apply to the present dataset due to 0% missingness.

What we added: A labelled subsection specifying: imputation method (none required), number of imputed months (0), and how missingness would be handled in rolling-origin training/evaluation under leakage-safe rules (deployment guidance).
Where: Section [Data preprocessing / Missing data handling], p. [4-5]; Table/Figure [T2; F1], p. [4;14].

Comment 5 (Hyperparameter choice is not fully audit-ready). Reviewer comment: The architecture/optimization/calibration steps are described, but the hyperparameter selection process is not fully audit-ready.

Response: We agree and expanded the description of how hyperparameters were chosen. We now state the search strategy (e.g., fixed a priori values vs. tuning), the tuning objective/metric, the validation scheme (time-respecting), the search space (ranges), and the final selected values. To make this auditable, we added a hyperparameter table and clarified how calibration (e.g., isotonic) was trained without leakage.

What we added: A "Hyperparameter selection" paragraph and a table listing final hyperparameters (and ranges/search where applicable).
Where: Section [BiLSTM methods / Training and tuning], p. [10]; Table [T1 or S2# Hyperparameters], p. [24-25].

Comment 6 (Additional experiment to demonstrate generalizability). Reviewer comment: Conduct an additional experiment to demonstrate the generalizability of the proposed model.

Response: We agree and added a generalizability check. Specifically, we performed an era-based evaluation by restricting scoring to a later period (e.g., 2021–2024) and recomputing aligned probabilistic metrics across models and horizons, which probes temporal transport under potential distribution shift. We report both performance summaries and (where feasible) significance testing on the restricted aligned subset.

What we added: Era-based (time-split) aligned evaluation and summary figure(s)/table(s).
Where: Section [Results: Generalizability across time / Era-based evaluation], p. [19]; Table [7-8], p. [21]; Figure [11-12], p. [19-20].
